# Qualitative analysis of anti-abortion discourse used in arguments for a 6-week abortion ban in South Carolina

**DOI:** 10.3389/fgwh.2023.1124132

**Published:** 2023-03-30

**Authors:** Victoria C. Lambert, Emily E. Hackworth, Deborah L. Billings

**Affiliations:** Department of Health Promotion, Education and Behavior, University of South Carolina, Columbia, SC, United States

**Keywords:** anti-abortion, legislation, policy, abortion rhetoric, abortion laws, pro-life movement, discourse, attitudes toward abortion

## Abstract

**Background:**

On June 24, 2022, The U.S. Supreme Court overturned Roe v. Wade, leaving abortion legislation entirely up to states. However, anti-abortion activists and legislators have organized for decades to prevent abortion access through restrictive state-level legislation. In 2019, South Carolina legislators proposed a bill criminalizing abortion after 6 weeks gestation, before most people know they are pregnant. The current study examines the anti-abortion rhetoric used in legislative hearings for this extreme abortion restriction in South Carolina. By examining the arguments used by anti-abortion proponents, we aim to expose their misalignment with public opinion on abortion and demonstrate that their main arguments are not supported by and often are counter to medical and scientific evidence.

**Methods:**

We qualitatively analyzed anti-abortion discourse used during legislative hearings of SC House Bill 3020, The South Carolina Fetal Heartbeat Protection from Abortion Act. Data came from publicly available videos of legislative hearings between March and November 2019, during which members of the public and legislators testified for and against the abortion ban. After the videos were transcribed, we thematically analyzed the testimonies using *a priori* and emergent coding.

**Results:**

Testifiers (Anti-abortion proponents) defended the ban using scientific disinformation and by citing advances in science to redefine “life.” A central argument was that a fetal “heartbeat” (i.e., cardiac activity) detected at 6 weeks gestation indicates life. Anti-abortion proponents used this to support their argument that the 6-week ban would “save lives.” Other core strategies compared anti-abortion advocacy to civil rights legislation, vilified supporters and providers of abortion, and framed people who get abortions as victims. Personhood language was used across strategies and was particularly prominent in pseudo-scientific arguments.

**Discussion:**

Abortion restrictions are detrimental to the health and wellbeing of people with the potential to become pregnant and to those who are pregnant. Efforts to defeat abortion bans must be grounded in a critical and deep understanding of anti-abortion strategies and tactics. Our results reveal that anti-abortion discourse is extremely inaccurate and harmful. These findings can be useful in developing effective approaches to countering anti-abortion rhetoric.

## 1. Introduction

On June 24, 2022, in a 5–4 decision, the Supreme Court of the United States overturned the constitutional right to abortion through the Dobbs v. Jackson Women's Health Organization decision, overturning the 1973 Roe v. Wade decision and leaving states to decide on and enact their own abortion legislation. Since then, abortion access has become increasingly difficult and confusing within a fragmented and polarized abortion landscape. Many states in which Republicans control state legislatures have passed extreme abortion restrictions or complete bans (1, 2).

Overturning the right to abortion granted by the 1973 Roe v. Wade decision was the result of decades of organizing by fundamentalist conservatives in the United States, who worked strategically over time through state-by-state actions to pass restrictive legislation. The past decade has seen a sharp rise in state abortion restrictions, with a record 108 state restrictions on abortion enacted in 2021 alone (3). At the same time, efforts to reverse antiquated legislation, known as trigger laws, were unsuccessful or not pursued, thereby making abortion illegal when Roe v. Wade was overturned (3).

Since 2011, multiple state legislatures have proposed or enacted legislation that bans abortion after a fetal “heartbeat” is detected (4). These bills promote misinformation that life is indicated by the detection of a “heartbeat” (more accurately described as electrical activity of cells) (5), which can occur as early as 6 weeks gestation, before most people know they are pregnant (6). A surge of 6-week abortion bans began in 2019 (7), with five states successfully enacting 6-week bans in 2019 alone, including Georgia, Kentucky, Louisiana, Mississippi, and Ohio (8).

Only one other study has analyzed the anti-abortion rhetoric around a 6-week abortion ban. Evans and Narasimhan conducted a narrative analysis of the legislative testimony around the 2019 6-week ban in the state of Georgia (9). They report that anti-abortion advocates in Georgia promoted fetal personhood and legal protection of fetuses by using “heartbeat” as a proxy for life. They also framed this protection as a matter of states' rights. Furthermore, these anti-abortion advocates misrepresented scientific findings and appropriated progressive successes, such as civil rights legislation (9).

In the current study, we replicate the approach used by Evans and Narasimhan to examine the anti-abortion rhetoric used by anti-abortion proponents in South Carolina (SC) in 2019, who aimed to pass a 6-week abortion ban. The South Carolina Fetal Heartbeat Protection from Abortion Act, House Bill 3020 (H.3020) was introduced to the SC House on January 8, 2019 (10). It required “testing for a detectable fetal heartbeat before an abortion is performed on a pregnant woman and to prohibit the performance of an abortion when a fetal heartbeat is detected.” Unlike the bill in Georgia, H.3020 did not pass in SC during the 2019 legislative session. However, a practically identical bill (S.1) was introduced to the SC state Senate during the 2021 legislative session, passed both the House and Senate, and was signed into law by Governor Henry McMaster on February 28, 2021. While it was initially struck down by a federal judge, this 6-week ban went into effect again soon after Roe v. Wade was overturned in June 2022. It was subsequently enjoined by the SC Supreme Court, which on January 5, 2023, ruled the 6-week ban was unconstitutional based on the right to privacy, which was added to the state Constitution in 1971 (11).

Roe v. Wade no longer exists to overrule state-level bans on abortion. In this historical moment, it is critical to deconstruct and understand the strategies and tactics that aim to restrict abortion access and to position these strategies within the context of a post-Roe world. Further, it is important to illuminate the consequences of restrictive abortion legislation, especially in terms of deepening abortion-related stigma and the detrimental impact on the health and lives of South Carolina residents, especially those marginalized by racism, poverty, and anti-LGBTQ and anti-immigrant sentiment and policies. By examining the arguments used by anti-abortion advocates, we aim to expose their misalignment with public opinion on abortion (12) and demonstrate that their main arguments are not supported by and often are counter to medical and scientific evidence.

## 2. Methods

### 2.1. Design and participants

We utilized publicly available videos of meetings of the SC House Judiciary Constitutional Law Subcommittee and the SC Senate Medical Affairs Subcommittee, during which members of the public provided testimony for H.3020. The meetings took place between March and September 2019, with video footage posted on the South Carolina legislature video archives website and the Women's Rights Empowerment Network Facebook page. We analyzed arguments from anti-abortion proponents, including 19 South Carolina legislative representatives as well as 22 community members who made anti-abortion arguments in support of the bill or demanding even stricter abortion legislation.

### 2.2. Procedure

Video files of the hearings were downloaded from the Women's Rights and Empowerment Network's Facebook page (https://www.facebook.com/WomensRightsandEmpowermentNetwork) and the South Carolina legislature video archives website (https://www.scstatehouse.gov/video/archives.php) and transcribed using Happy Scribe, a virtual transcription service. The transcripts were then fidelity checked by the research team and imported into NVivo, where they were thematically coded. Researcher-perceived demographic characteristics (e.g., gender, age, race) of the testifiers were noted when testifiers did not provide characteristics in their testimony. We also conducted a word count of terminology used in the testimonies to characterize fetuses.

We used a combination of *a priori* and emergent codes. The *a priori* codes were developed based on a codebook from a previous analysis of Georgia's “heartbeat” bill hearings, conducted by Evans and Narasimhan (9). Authors 1 and 2 began by independently double-coding each transcript. After initial coding of each transcript, a coding comparison query was conducted to assess coder agreement for each code. Agreement ranged from 83%-100% across all transcripts.

We implemented a constant comparison approach, where we coded the data then paused to review the codebook, examples of individual codes, and overlap between the codes. All three authors met to discuss the codes after each initial coding of a transcript. We reviewed coding of themes for which there was lower reliability (i.e., 90% or lower) and clarified any questions about the codebook that arose while coding. We also discussed any new codes or other changes we thought should be made to the codebook. When changes were made to the codebook, each of the previously coded transcripts was recoded by one of the first two authors based on extensive discussion about the codes among all three researchers. Additionally, the research team regularly communicated with colleague researchers Evans and Narasimhan, who analyzed 6-week ban hearings in Georgia. Through this process, the codebook was frequently reevaluated and refined to best represent the South Carolina data.

We expanded the codebook used in the analysis of Georgia's 6-week ban to include new themes that arose in South Carolina. First, we added codes to characterize scientific misinformation, including codes identifying different types of evidence (e.g., anecdotal, statistics, quotes, health professional credentials) and codes around specific arguments used to promote misinformation (e.g., abortion is harmful, advances in science and technology). We also added codes that characterized moral arguments against abortion, including limiting government overreach, racism, abortion clinic profit, carry to term coercion, abortions for convenience or burden, equating abortion with murder, organized responses to abortion, and value of life. Finally, we expanded a single code for rape used in the Georgia codebook by adding more specific codes, such as arguments for and against exceptions for rape, abortion as evidence of rape, descriptions of rape victims, and responsibility of rape victims to report.

### 2.3. Study ethics

The Institutional Review Board at the University of South Carolina reviewed the study protocol and determined this study did not meet the criteria for human subjects research. Although quotes can be matched with video footage, throughout this article, we do not directly identify any individual who provided testimony except the primary legislative sponsor. Given that all testimony analyzed is part of the public record, our approach respects research ethics related to privacy and confidentiality.

## 3. Results

Researcher-perceived and self-identified characteristics of the anti-abortion proponents who gave testimony are summarized in Table 1. We analyzed testimonies from 41 individuals, including 19 SC legislators, four physicians or nurses, six anti-abortion advocates, six religious figures, two crisis pregnancy center employees, one student, and four people who did not provide their occupation in their testimony. Twenty of the anti-abortion proponents who testified identified as “Christian” as part of their testimony. While we were unable to obtain self-identified socio-demographic information about the community members who testified, we estimated most to be 30–54 years old (*n* = 12) or 55 or older (*n* = 16), white (*n* = 34), and male (*n* = 28).^1^

**Table 1:** Perceived characteristics of anti-abortion speakers at 2019 H.3020 hearings.

Variable		# of Participants (*N* = 41)
Age	18–29	3
	30–54	12
	55+	16
	Unsure	10
Gender	Male	28
	Female	13
Race	White	34
	Black	7
Occupation (self-identified)	Government Figure	19
	Physician or Nurse	4
	Anti-Abortion Advocate	6
	Religious Figure	6
	Crisis Pregnancy Center Employee	2
	Student	1
	Other	4
Religion (self-identified)	Christian	20
	Unknown	21

### 3.1. Argument frames

In our analysis, we found that arguments could be classified into two major argument frames: scientific disinformation and moral arguments, with some anti-abortion proponents using both frames to build their case for supporting H.3020.

### 3.2. Scientific disinformation

Throughout the testimonies, proponents of H.3020 framed arguments using claims based in scientific disinformation. These types of claims misrepresented scientific findings and used scientific and medical terms and explanations of pregnancy and abortion in inaccurate or misleading ways to justify the ban. Both individuals with medical backgrounds and those without scientific training used scientific arguments to support the ban. Arguments that relied on scientific disinformation can be classified into four themes: (1) Arguments from biased medical professionals; (2) Arguments that misrepresented science; (3) Arguments that attempted to redefine life from a scientific perspective; and (4) Arguments using value or logic statements to connect scientific and moral arguments.

#### 3.2.1. Medical professionals supporting the ban were biased and used coercion

Four supporters of H.3020 from the 2019 hearings were medical professionals. Each began their statements by describing their training and credentials in detail, with only one claiming to specialize in obstetrics. The other three mentioned that their training in obstetrics was outdated or not extensive, yet they felt they could serve as experts despite their “limited experience.” One even stated, “I don't have the level of expertise and experience of my obstetrical colleagues,” yet continued to share his testimony about refusing to refer pregnant people seeking abortions to abortion providers. All the medical professionals mentioned the Hippocratic Oath, referring to it as their “oath to protect the life of the born and the unborn,” and their duty “to speak on behalf of the unborn.”

The medical professionals with experience working in family medicine or obstetrics shared stories of coercing pregnant people to listen to and view their ultrasounds.^2^ One stated:

“We have a look at the ultrasound and have the mother see the heartbeat. Upon seeing the heartbeat, she realizes this is a child and it's her child … Most of the women who have an opportunity simply to see truth—that this is their child they’re carrying—decide to continue with the pregnancy.”

In a later testimony, this same physician stated that he would “never coerce,” yet he described how he would offer free ultrasounds to pregnant people who had made it clear they wanted an abortion and then use that ultrasound as an opportunity to convince them to continue their pregnancies:

“We'd put the ultrasound probe on and see this teeny little peanut of a fetus—or an embryo, really– and then hung around a little bit further and then see this teeny little fluttering, which comprised a kind of a rapid beat that you could see. And almost invariably– not all the time, but almost invariably– I would point that out and the lady would say, ‘Is that my baby's heartbeat?’ Almost invariably, that was a comment that was made. … And then after that, the vast majority of those women who would see that heartbeat would then decide to keep their pregnancies. Not all, but many of them did decide to keep them.”

Medical professionals also used medical terminology in biased ways to bolster their claims. One described fetal development by saying:

“At 6 1/2 weeks, the teeny little baby is about a little bit less than an inch long; has very incipient eyes, head, chest, a two chambered heart; has limb buds with teeny little buds that will eventually become fingers at the time of the average time for elective abortion.”

Another physician stated, “We can discuss all day long here today whether the fluttering, the pulsation and cardinal vessels of a tiny embryo comprise a heartbeat or two chamber for chamber heart.” Despite his medical expertise, he went on to say, “When a mother says that it is a heartbeat, that's a heartbeat.”

Intertwining their anti-abortion opinions with their medical “expertise” and “experience,” the medical professionals who testified in support of the 6-week ban made grandiose claims about how wrong they believe abortion to be. One stated, “Any medical procedure that interrupts or terminates that developing human life for whatever reason constitutes the taking of a human life.” Another said:

“There is no circumstance, no matter how desperate or difficult, that justifies the killing of innocent unborn children with beating hearts. And trust me, as a family doctor, I have dealt with all of these desperate and difficult circumstances that surround the conception of unborn children.”

Another obstetrician and medical director at a crisis pregnancy center stated, “I have lots of patients and I don't tell them, ‘I’m a Christian, I don’t believe in abortion, you can’t have an abortion’ … I talk to them about their options.”

#### 3.2.2. Anti-abortion proponents misrepresented science

Many anti-abortion proponents relied heavily on data, statistics, or numbers to bolster their claims. However, they rarely referred to actual sources and often misrepresented the statistics they cited. Representative John McCravy, the primary legislative sponsor of H.3020 stated, “If that heartbeat's detected, there's a 90 percent chance of that baby's survival” and “we know from medical science that that's about 90 to 95 percent now.” Fetal cardiac activity – what supporters of this bill call a “heartbeat” – can be detected as early as 6 weeks gestation, which is long before the possibility of a viable birth. While viability is a complex medical concept determined by more than gestational age, leading experts in the field of fetal and maternal medicine do not consider births before 20 weeks gestation to be near the threshold of viability (i.e., periviable) (13). Furthermore, the American College of Obstetricians and Gynecologists reports that 95% of births before 23 weeks gestation result in fetal death (14).

Other speakers made erroneous statements about the consequences of abortion, such as “the statistics show that 10% of women who have abortions never have another child” and “there is no data to support the claim that abortion is safer than childbirth.” These speakers did not provide sources for these claims. However, even if the former statement were true, 90% of people who get abortions would go on to have a child, which is high given the fact that nearly 17% of older adults in the United States do not have biological children (15). There are not reliable studies that follow people for the rest of their reproductive years to ascertain if they have a biological child after getting an abortion; however, most people who have abortions already have at least one child (16). Furthermore, compared to people denied an abortion, people who receive an abortion are more likely to become pregnant again in the subsequent 5 years (17). Regarding the safety of abortions, many studies have found that legal abortions are much safer than childbirth (18–20). Considering mortality alone, abortions are significantly safer than giving birth. In the United States in 2019, there were 20.1 deaths per 100,000 live births (21). In contrast, the rate of abortion-related deaths from 2013 to 2017 was 0.44 legal induced abortion-related deaths per 100,000 reported legal abortions (22).

Some speakers used anecdotal evidence to generalize about the experiences of patients, displaying that they lacked the ability (or desire) to differentiate between their subjective experiences and scientific evidence. A registered nurse referred to a “brief survey” that she conducted of obstetrics and gynecology (OB-GYNs) at the hospital where she worked. She said her sample consisted of “a handful of doctors” but she used research terminology and drew generalized conclusions. She stated that, “All but one [doctor] admitted to seeing multiple women in the emergency room with abortion complications,” and that there were “frequent diagnoses of retained products of conception, punctured uterus and undiagnosed ectopic pregnancy, a life-threatening condition.” She followed up by stating, “That is when a woman has had an abortion without having an ultrasound to determine the location of the pregnancy.” Similarly, an obstetrician, who was also the medical director at a crisis pregnancy center, claimed to see patients who reported physical and mental harm from abortions. Another medical doctor recounted his experience with patients seeking abortions, stating, “The vast majority of those women who would see that heartbeat would then decide to keep their pregnancies.” These speakers misrepresented anecdotal experience as scientific evidence, describing experiences that contradict research showing that abortions are very safe (18–20, 23) and that most people do not regret getting them (24).

Disregard for scientific evidence was also demonstrated when anti-abortion proponents stated that the scientific backing for the bill was obvious without providing data or evidence to support this claim. For example, one person stated, “Obviously, the scientific case for life has been made. We've all been through middle school [and] high school biology.” He went on to say, “We know that organisms that reproduce sexually when the sperm fertilized egg, there's a unique, genetically unique organism.”

Table 2 outlines the most common false claims made by anti-abortion proponents as well as scientific evidence refuting these claims.

**Table 2:** Scientific and medical disinformation.

Examples of false claims^a^	Evidence refuting these claims
Describing fetal cardiac activity as a “heartbeat”	Cardiac activity occurs long before the heart is developed (25).
Fetal cardiac activity ensures viability and “survivability” (confounding viability with risk of miscarriage)	While viability is determined by more than gestational age, experts in the field of fetal and maternal medicine commonly consider the point of viability to occur between 23–24 weeks gestation (14, 26). Futhermore, periviable births, those near the limit of viability, are those that occur from 20 0/7 weeks to 25 6/7 weeks of gestation (26).
Abortions are harmful/more dangerous than giving birth	Legal abortions are extremely safe (18–20, 23). Risk of mortality from giving birth is 14× greater than for abortion (27).
Abortions prevent later pregnancies	Abortions have no effect on fertility (23). They also appear to enable people to have later intended pregnancies. In a study following people 5 years after seeking an abortion, people who were able to get an abortion had higher overall pregnancy rates and higher intended pregnancy rates compared to people denied an abortion (17).

^a^ Sources of these claims were not mentioned or were based on anecdotal evidence.

#### 3.2.3. Anti-abortion proponents attempted to redefine life and personhood

Another prominent strategy of anti-abortion proponents was attempting to redefine life and personhood from an ostensibly scientific perspective. One way they did this was through use of various words and phrases to promote a new standard of life and personhood. Throughout all the anti-abortion testimonies we analyzed, the term “heartbeat” was used 97 times when not referring to the bill itself; “baby” was used 62 times; “unborn” was used 45 times; “unborn child” was used 16 times; “personhood” was used eight times; and “pre-born” was used six times.

The word “heartbeat” was particularly instrumental to the anti-abortion arguments trying to redefine life from a scientific perspective, with many supporters of the bill framing their arguments around the heartbeat being the new standard for life. The primary legislative sponsor of the bill stated in his opening testimony, “…the heartbeat is a definite marker and a predictability of survival.” One pastor stated, “While not the beginning of life, the heartbeat is a universally recognized indicator of life.” Another speaker, the executive director of an anti-abortion non-profit, stated, “The absence of a heartbeat indicates death, then logically the presence of a heartbeat indicates life.”

Anti-abortion proponents also suggested that advances in science and technology have changed the standard for fetal viability and how the medical field understands life. For example, representative McCravy, H.3020's primary legislative sponsor, stated:

“There's also a consensus among medical experts, scientists, lawyers and ethicists that the standard of viability has changed. That we now know many more things about the unborn child that we did not know in the 1970s. So we've had so many advances in medical and scientific technology that have expanded our knowledge of prenatal life.”

Neither this representative nor anyone else making similar arguments cited evidence for the claim that advances in scientific technology had redefined the medical standards of viability or life. However, though there have been advances in care for pre-term deliveries, fetal viability occurs long after 6 weeks gestation. Births between 20 and 26 weeks are considered “periviable,” with viability depending on various factors surrounding a pregnancy (13).

In contrast to those who tried to redefine life and personhood at a fetal “heartbeat,” some anti-abortion proponents refused to use this as the new standard for life. For example, a medical professional in favor of the bill stated, “My expert medical testimony is that human life begins at conception. That was what I was told in medical school 40 years ago. That was true then. That's true now. Nothing has changed in the ensuing 40 years.” One anti-abortion proponent, who opposed the bill in favor of stricter legislation, stated:

“The heartbeat bill does not establish justice for all human beings at fertilization but chooses the biological benchmark which may occur and be detected for a month and a half or later after fertilization and allows all human beings in the womb prior to that point to be exterminated.”

#### 3.2.4. Anti-abortion proponents used value or logic statements to connect science and morality

Many supporters of H.3020 intertwined scientific claims with value or logic statements as the basis of moral arguments. Since science provided a new standard for detecting life, anti-abortion proponents argued it was clear that abortion after a “heartbeat” is detected is taking a life, which is immoral. Some who used this line of reasoning referred to it as “logical,” “rational,” or “common sense” when making their arguments. A pastor said, “Science and common sense tells us that a heartbeat signifies life,” while not following up with an explanation of what he meant by either “science” or “common sense.” Another speaker, a family physician, stated:

“You are logical and rational people, and I trust that you make your decisions based on good rationale and good logic and not on emotion. Life begins at conception and there is a heartbeat in every unborn child. Nothing changes that fact. That's logical, that's rational. And I trust that you make your decisions based on good logic and good rationale.”

The framing of H.3020 as the heartbeat bill provided an opportunity for anti-abortion advocates to claim a novel scientific standard for life, which made it easier for supporters of H.3020 to claim that their connection of scientific and moral arguments was logical.

### 3.3. Moral arguments

Supporters of H.3020 made various arguments that framed abortion and those providing or seeking abortions as immoral. Moral arguments across the testimonies could be classified into five themes: (1) Promoting the (perceived) righteous cause of protecting the unborn; (2) Religion as the basis of morality; (3) Vilifying and stigmatizing the pro-choice movement; (4) Vilifying healthcare providers; and (5) Vilifying and stigmatizing people who get abortions.

#### 3.3.1. Anti-abortion proponents promoted the perceived righteous cause of “protecting the unborn”

Based on the assumptions that a “heartbeat”– or some other indicator of fetal development– signified human life, anti-abortion proponents argued that abortion after 6 weeks is murder. While many simply used the word murder (or related terms) to describe abortion, others attempted to explain why they believed abortion was equivalent to killing a person. Some supporters of the bill believed that life begins at conception and, hence, abortion before 6 weeks gestation should also be defined as murder. However, many argued that, since absence of a heartbeat is an indication of death, presence of a heartbeat indicates life. Hence, in “civilized” societies, abortion after detection of a fetal “heartbeat” is taking a life. Many promoted the fetal “heartbeat” as the new standard for life in a modern or “civilized society” that does not allow innocent humans to be killed. One person who provided testimony, the director of an anti-abortion non-profit, summarized this line of thinking:

“Another fact-based saying is if the absence of a heartbeat indicates death, then logically the presence of a heartbeat indicates life. In a civilized society, we should all agree that the human heartbeat is the objective scientific proof of life. In a civilized culture, we can all agree that it is barbaric and a savage act to kill an innocent, innocent member of the human family with a beating heart.”

Supporters of the bill called abortion genocide and both directly and indirectly compared it to historical injustices and atrocities. In their view, just as society's morals have progressed regarding historical atrocities, society must progress by adopting this new standard of life–the fetal “heartbeat.” One white male proponent of the bill said the following:

“If nothing else, history tends to judge people harshly based on the standards of the current day. Not at the time of the actual event. I never hear anyone try to justify slavery based on the standards of today. Women not having the right to vote seems ludicrous. The Jim Crow laws of the South seem cruel by today's standards. But what will we tell our grandkids about abortion when they ask why we had the chance to stop it? But we fell prey to political pressure and let the carnage continue. How would they judge us? Some will justify it as a right the mother has to do with her body as she wants, never once considering the future mother she may be carrying.”

To further demonstrate the morality of H.3020 and its advocates, supporters of the bill framed the unborn as a targeted and vulnerable group in need of special protections. To do this, they co-opted language and ideas from human rights movements, suggesting abortion is an inhumane practice that violates universal principals of morality. Human rights language, including terms such as “intrinsic value” and “dignity of human life,” was used to extend the rights of humans to embryos (which anti-abortion proponents refer to as “fetuses”). Thus, supporters framed this bill and its advocates as the true champions of morality and human rights. Indeed, many supporters touted their own commitment to the “value of life.” They urged representatives listening to do the same, adding that they believed the government is responsible for protecting life through banning abortions. One speaker co-opted human rights language by saying:

 “Unlike pro-abortion advocates, the pro-life movement firmly believes in the value and dignity of each human life. That includes every single person in this room. And I want to tell each of you in this room, your life matters. Your life has intrinsic and immeasurable value because you are human.”

A legislative representative more explicitly framed abortion as a human rights issue:

“The U.S. Constitution, which all our laws must pursue, states in the Fourteenth Amendment: No state shall deprive any person of life without due process of law and order, not to any person within its jurisdiction equal protection of the laws regardless of the age, size, or dependent status. A child with a heartbeat should be treated with dignity. These unborn men and unborn women have human rights too. And yes, even children conceived through the terrible crime of rape have a right to life and justice. They have a right not to have their bodies mutilated, their choices eliminated, and their heartbeats stopped. I stand against abortion so passionately because it is the chief example in our time of the callous violation of human rights. It is our job in government to defend against this. Human children deserve to be defended.”

#### 3.3.2. Anti-abortion proponents used religion as the basis of morality

While religious rhetoric was sparse across the testimonies, some anti-abortion proponents used their primarily evangelical Christian religious beliefs as the foundation for their moral arguments about the value and definition of life. For example, speakers stated they supported the bill because they believed in the value and sanctity of life of the unborn, a belief informed by their religion. In addition to claiming to respect the “value of life,” speakers making religious arguments used multiple adjectives with moral implications to describe life, such as “precious,” “sacred,” and “dignity.” The following quote exemplifies many of the adjectives used to discuss their view on the “value of life” as well as the seamless connection speakers made between religious beliefs and morality:

“We want the heartbeat bill because we want to protect the God-given value and dignity of all human life, including our own. Unlike pro-abortion advocates, the pro-life movement firmly believes in the value and dignity of each human life. … Your life has intrinsic and immeasurable value because you are human. If we believe we can discard human life at its most helpless and most vulnerable, it cheapens the innate and immeasurable value that God bestowed upon each one of us at that very first moment of our own existence. Tiny, precious, and innocent lives are at stake in this legislation. But so is our very own worth and dignity.”

Anti-abortion proponents who discussed religion also used other tactics to connect their religious beliefs to the immorality of abortion. Some quoted biblical scripture as evidence that “life” in the womb is determined and created by God, with some speakers explicitly stating that life begins at conception. Others used anecdotes and personal stories. Some of these stories were about pregnant people who considered abortion yet decided to carry to term because of their religious beliefs. Other stories centered people whose mothers considered aborting them but did not. While religious speakers described the inherent value of “pre-born” life as bestowed by God in scripture, they often tied the worth of the people in these stories to their potential to advance Christianity.

#### 3.3.3. Anti-abortion proponents vilified the pro-choice movement

In contrast to their portrayals of themselves and other advocates of H.3020, supporters of the bill framed the pro-choice movement and opponents of the bill as immoral. They framed the unborn as a vulnerable group, thereby claiming that pro-choice advocates were not only discriminatory but also proponents of child sacrifice and genocide. They further claimed pro-choice advocates devalue life and human rights and strategically dehumanize the unborn to achieve their goals. One supporter of the bill—a male physician and medical director for a crisis pregnancy center—compared societal approval of abortion to historical acceptance of chattel slavery and the Holocaust.

“So you think about throughout history to make something to be able to commit atrocities against a group of people. What society has done is dehumanized them. United States with slavery and Nazi Germany with the Jews. That's what they did. They said, 'Well, they're not human', right? That's what we did. Right. Shameful. That's what we're doing. That's what we're doing with the unborn child. … I think this is a blight on our nation. And I think there's a genocide, to which we're all going to be called by our maker.”

#### 3.3.4. Anti-abortion proponents vilified healthcare providers who perform abortions

Supporters of the bill also vilified healthcare providers and clinics providing abortions, claiming they make exorbitant incomes from abortions while providing inadequate care and coercing people into getting abortions. One pastor questioned the motives and morality of a nearby healthcare provider offering abortions:

“This past weekend, I was at [name of medical center], which is one of the busiest abortion clinics in the southeast. One of the reasons it is, is because it's the cheapest. … But I quickly asked, how much money do they make off this one center? They own three. And the answer is four to five million dollars a year. And I was thinking, man, are they really here for women? Or are they here to line their pockets? …  Our battle is against the evil. Our battle is against not anybody that's against this bill. Our battle is for flesh and blood. It's not against flesh and blood.”

Another speaker—a physician and medical director at a crisis pregnancy center—attacked Planned Parenthood:

“And one of the main reasons we're here today is money. This is money. And power. This is a multi-billion dollar business, abortion. And I'm telling you, I have so many patients who come to me and said ‘they didn't tell me about the risk of depression. They didn't tell me the risk I would get a perforated uterus and have a hysterectomy.’ I've seen that happen. ‘They didn't tell me about any of the risk of the suicidal thoughts and suicidal ideation.’ I have family members who have had an abortion who've never gotten over the depression from that. So we need to–if we're going to provide safe abortion–we need to make sure that people like Planned Parenthood who are making billions of dollars—half a billion of our tax dollars are going to Planned Parenthood– we need to make sure that they are properly counseling women on the not only physical risk of abortion, but the mental risk.”

In 2019, Planned Parenthood's total revenue, including both government funding and private donations, was $1.6 billion while its expenses were $1.5 billion (28). However, Planned Parenthood is prohibited from using any federal funding it receives on abortion services, and abortions account for only 3% of the total medical services provided at Planned Parenthood health centers (28). Furthermore, there is ample evidence that abortions are safer than childbirth (18, 19) and other common medical procedures (29) and that people seeking an abortion who are able to get one fair better in terms of their short-term mental health (30) and longer-term physical health and financial well-being than people denied abortions (31).

#### 3.3.5. Anti-abortion proponents vilified people who receive abortions

Anti-abortion proponents used multiple tactics to both stigmatize abortion and malign people who receive abortions. They characterized people who receive abortions as lazy and irresponsible or selfish and immoral and stated their objections to “abortions of convenience,” defined by multiple speakers as having an abortion for social or economic reasons and not for the life of the pregnant person or because of rape or incest. While many of these speakers stated or implied that most abortions are done for “convenience,” none cited evidence for this claim.

Supporters of the bill described their aversion to “abortions of convenience” with statements like this from a legislative representative:

“Over 90% of the abortions that are done in the United States are what? They are done for the manner of convenience. Not because of life of the mother. Not because a rape or incest. But because of convenience issues.”

Another example came from a testifier who was an educator at a Christian university and the president of an anti-abortion non-profit: “The vast majority of abortions are obtained for social and economic reasons, not rape, incest, or life of mother. … Studies indicate that over 90% of women seek abortion for social and economic reasons.”

Some anti-abortion proponents who described people who get abortions as selfish also described them as victims. They claimed abortions are mentally, emotionally, and physically harmful to people who receive them. For example, one person described her own abortion as selfish, yet said that she and other people who get abortions are unaware of (what she perceived as) emotional repercussions of abortion:

“I got an abortion, and it was a heartless, selfish act of snuffing out a precious life for my convenience. Women don't know, nor are they told about, the guilt and the shame that they will carry the rest of their lives. Abortion doesn't just end the life of a baby. It hurts women, and it scars them for life. I have heard hundreds of testimonies, and I know many women will deny what I just said. They claim there have been no repercussions. However, even if they hide their guilt and their shame from themselves, or they deny it or bury it so deep that they don't feel anything anymore, they still live with it the rest of their lives.”

In contrast to this quote, researchers have found that, among people seeking abortions, those *denied* an abortion are more likely in the short-term to experience adverse mental health outcomes, such as anxiety, low self-esteem, and low life satisfaction compared to people who receive abortions (30). The person who said the previous quote went on to describe her husband's disdain for her past abortion, revealing the potential source of the guilt she felt for getting an abortion:

“Yesterday, my husband said to me, ‘You can be forgiven, but you have still taken a life.’ Heartless. For women who have had an abortion, those are fighting words because it's difficult to face the reality that we have taken a life. Abortion is a heartless act and the argument that women have a right to their own bodies is a smokescreen to hide the reality that they can't face.”

Another anti-abortion proponent who was an educator at a Christian university and the president of an anti-abortion non-profit implied that people who get abortions are victims because healthcare providers do not inform them of the risks of having an abortion. She used this argument to advocate for the new bill, which would require informed consent and provision of information about the “harms” of abortion to anyone seeking an abortion:

“For many of these women, abortion is not an act of liberation. Rather, it is a violent act of despair. Many of these women have indicated they would carry their child if they were not abandoned by the father, pressured by employers, or rejected by parents. Further, many women in the United States, unlike those in South Carolina, have not been guaranteed informed consent. … They undergo abortions with no knowledge of fetal development, or knowledge of alternatives, or knowledge of the risk involved. For many women, abortion is a skillfully marketed product to prey on the fears of women in crisis. When women discover the physical, mental, and emotional scars that often surface, it is too late. I’ve met many of these women myself.”

As demonstrated in the quote above, supporters of the bill framed people who get abortions as desperate victims by associating abortions with abuse and coercion. In legislative sessions on the inclusion of an exception in the bill for rape and incest, speakers focused more intently on actual victims of abuse seeking abortions. Some against the exception claimed abortion protects rapists because a baby is evidence of rape and that abortion perpetuates the trauma of victims. Conversely, they claimed that carrying a pregnancy to term provides deliverance, healing, and vindication to victims of rape and incest. This discourse around victims of rape, incest, and coercion served to stigmatize abortion as associated with abuse. A quote from one speaker–the founder and president of an anti-abortion organization–exemplifies this discourse:

“And the younger a girl is, the more likely it's someone in her household, family member who's been raping her. And the more likely that it's been going on for years. And guess who reveals the rape? The baby. Her baby is ultimately her hero who can deliver her out of that abusive situation. But we see time and time again how oftentimes it's her own mother who's trafficking her, who takes her to the abortion clinic where they cover up the rape and then send her right back for repeated abortion, after abortion, after abortion. … And it is absolutely absurd to suggest that somehow more violence brings healing, more violence in the exact place where she was traumatized is somehow going to bring healing. But babies do have a way of bringing healing. … And I am so concerned when people say that somehow, you know, she's going to be better off after an abortion, when studies show that she's four times more likely to die within the next year after the abortion. They have a higher rate of murder, suicide, overdose because, again, more violence doesn't bring healing”

As stated in previous sections, there is no evidence that abortions are associated with negative mental health outcomes. Indeed, people who are denied an abortion are more likely to experience poor mental health than people who get an abortion (30).

## 4. Discussion

Our analysis of legislative testimony in support of H.3020, both by SC legislative representatives and the public, reveals that anti-abortion advocates primarily used scientific disinformation and moral arguments–based in their concepts of morality–to promote this bill. The only prior study to investigate anti-abortion arguments used in legislative hearings for a 6-week abortion ban came out of Georgia's 6-week ban, which was introduced in 2019, around the same time as SC H.3020. Many of the anti-abortion arguments and tactics we found in South Carolina were similar to those reported in the analysis of Georgia's ban (9), including misrepresenting scientific and medical findings, redefining life and personhood with the use of “fetal heartbeat” language, and comparisons of abortion to historical atrocities while framing opposition to abortion as defending human rights. However, anti-abortion proponents in South Carolina employed several arguments and strategies that were not found in the analysis of legislative hearings in Georgia. Findings unique to South Carolina included descriptions of biased and coercive care from medical professionals, use of value and logic statements based on scientific disinformation, use of religious rhetoric, and vilification of healthcare professionals and people seeking abortions.

In the current study, we found that blatant misrepresentation of science was commonly employed, with some speakers claiming there were scientific justifications for the ban without explaining these justifications and other speakers presenting opinions and anecdotal evidence as factual. This emphasis on medical and scientific arguments is consistent with anti-abortion arguments reported in an analysis of Georgia's 6-week ban. However, prior studies have found an historic lack of science-based arguments from anti-abortion activists, even within the last decade (32). The framing of this legislation as the “heartbeat bill” and the related scientific rationale used to ban abortions at 6 weeks may have allowed advocates of abortion restrictions to expand their rhetorical strategies by incorporating more scientific framing.

Though their scientific framing may have been relatively novel, anti-abortion advocates in the current study used scientific arguments as the basis for anti-abortion moral arguments that have long been used to promote abortion restrictions. Moral framing was prominent in the current analysis, with defenders of the bill providing arguments for why abortion was immoral and banning abortion was moral. This finding reflects a recent analysis of legislative discourse about anti-abortion policies, which found that morality frames were more common in discourse on abortion bans compared to discourse on other types of abortion restrictions (32).

The claim that fetal cardiac activity–which supporters of SC H.3020 inaccurately called a “heartbeat”–indicates life was fundamental to many of the moral arguments used by anti-abortion speakers. Using cardiac activity as a proxy for life and personhood, defenders of the ban asserted that abortion after detection of fetal cardiac activity is murder. Supporters of a similar 6-week abortion ban in Georgia in 2019 also used cardiac activity as a proxy for life and personhood (9). Advocates of abortion restrictions have long defended fetal personhood and “right to life” and claimed that abortion is murder (32). However, this novel and extreme legislation–deceptively named a “heartbeat bill”–gave anti-abortion advocates a new way to frame fetal personhood and connect supposedly scientific and moral arguments.

Anti-abortion advocates also endorsed the so-called morality of the 6-week ban by co-opting civil and human rights language and explicitly comparing their efforts to eradicate abortion to historical civil and human rights movements. This rhetorical strategy was also prominent in an analysis of public testimonies in support of a similar 6-week abortion ban in Georgia in 2019 (9). Co-option of and comparison to progressive, including civil rights, discourse have become common tactics in the anti-abortion space over the past decade (33, 34). Prior studies have found that anti-abortion organizations and advocates appropriate the language of social justice organizations and movements, such as Black Lives Matter; they also frame abortion restrictions as moral by comparing abortion to slavery, eugenics, and genocide and equating their advocacy to the historical efforts to eradicate those atrocities (33, 34).

Another tactic used by anti-abortion speakers in the current study was to frame the ban as sensible legislation by using phrases like “logical” and “common sense.” This type of language was not found in anti-abortion testimony for Georgia's 6-week ban (9). However, similar language was used during a congressional hearing for the 2014 federal Women's Health Protection Act, during which anti-abortion senators defended state abortion restrictions by referring to them as “common sense” legislation and claiming a majority of American people supported the restrictions (35). In an analysis of those hearings, Duffy (2015) argued that use of “common sense” language appeals to populist ideals by framing abortion restrictions as policies supported by and aligned with the values of sensible, average Americans. This rhetoric thus positions those opposing the “common sense” policies (in this case, abortion restrictions) as enemies of the American people. Duffy contends that this populist rhetoric in abortion policy discourse masks the damaging effects of anti-abortion legislation by focusing on conventional conservative talking points (e.g., arguments around states' rights and federal government overreach) rather than the actual impact of these bans on people's health (35).

Another theme in the current analysis that was not found in anti-abortion arguments for Georgia's 6-week ban (9) was negative portrayals of people involved in abortions, including providers and patients. Foundational to these negative portrayals was the claim that abortions are dangerous, which has been a common claim among U.S. anti-abortion advocates at least since the U.S. Supreme Court's Casey v. Planned Parenthood decision in 1992 (32, 36). Anti-abortion proponents have used this false claim to frame abortion restrictions as benefitting rather than harming women (32, 36–38). Claiming that abortions were harmful allowed proponents of the 6-week ban in South Carolina to both argue that abortion providers were deceiving patients about the risks of abortion and that abortion patients were being victimized.

Since the U.S. Supreme Court's Casey v. Planned Parenthood decision in 1992, abortion opponents have strategically vilified abortion providers, claiming that they mislead people about the risks of abortion (36). This theme remains prominent in anti-abortion discourse today (33, 37). In the current study, we found explicit negative portrayals of abortion providers, with anti-abortion proponents endorsing inaccurate claims about the physical and psychological consequences of abortion and claiming that abortion providers were nefarious for not disclosing these supposed harms and for ostensibly profiting off people who get abortions. In contrast, anti-abortion healthcare providers who testified in support of SC H.3020 described situations in which they provided biased or coercive care to patients seeking abortions. This finding suggests anti-abortion medical professionals are the providers who are actually harming patients.

In the current study, anti-abortion proponents making moral arguments also vilified people who get abortions by describing them as either lazy, selfish, and immoral or desperate victims of poverty, abusive relationships, and healthcare providers. They claimed these people are duped or forced into having abortions. Some speakers also claimed that abortions further traumatized victims of abuse, leading to a downward spiral of other harmful behaviors, including drug use and suicide. This discourse on the circumstances and effects of abortion both stigmatized abortion and allowed supporters of the abortion ban to claim it would protect women.

Prior research has reported that anti-abortion advocates commonly promote lies about the harms of abortion in order to claim that abortion restrictions protect women, with some even declaring abortion opposition as a feminist stance (32, 36, 37). This rhetoric is part of what has been termed “mother-child” framing, a relatively recent strategy through which abortion opponents assert abortion restrictions promote the wellbeing of both babies (fetuses) and pregnant people (36, 37). Claims that abortion restrictions protect women, are feminist, and are compatible with the wellbeing of mother and child have become more common in the post-Casey era (36). Before the 1992 Casey decision, anti-abortion proponents explicitly promoted fetal rights over the bodily autonomy of pregnant people; however, in recent decades, they have adopted a more “pro-woman” approach, in part to make abortion restrictions seem less radical (36–38). The recent rise of extreme abortion bans and the rhetoric promoting them (e.g., fetal “heartbeat” language) may suggest that anti-abortion strategies are regressing, with a renewed emphasis on fetal rights and personhood (37). Indeed, the current study and the analysis of anti-abortion rhetoric around Georgia's 6-week ban (9) suggest that personhood rhetoric is becoming more prominent among advocates of extreme abortion bans.

Claims that abortions are dangerous are false. Legal abortions are much safer than childbirth (18, 19) and other common medical procedures (29). Furthermore, people seeking an abortion who are able to get one experience better short-term mental health (30) and longer-term physical health (20) and financial well-being (39) than people denied abortions. Negative experiences related to abortion (which appear to be infrequent) can likely be attributed to inequitable social systems that oppress the most vulnerable abortion patients and not due to abortion itself (40, 41).

Religious arguments in the current study were sparse yet notable given their absence from anti-abortion arguments in legislative hearings for Georgia's 6-week abortion ban (9). In South Carolina, anti-abortion proponents used religion as the foundation of personal and societal morals by expressing how much they valued “life” in the womb, often tying this value and their definition of life to their Christian faith. Recent studies are mixed on the prominence of religious rhetoric in anti-abortion discourse. A 2013 analysis of anti-abortion discourse from anti-abortion forums, organizations, and parliamentary representatives in Canada found that religious arguments were not common (38); indeed, activists explicitly discouraged people from making religious arguments to support abortion restrictions, and scientific arguments appear to have replaced religious arguments for the basis of fetal personhood arguments (38). However, this analysis from Canada is dated, and the abortion landscape in Canada differs substantially from that in the United States. A more recent study of abortion discourse on Twitter found religious arguments were prominent in anti-abortion Tweets (42). Hence, the prominence of religious discourse may vary by platform. Additional investigation of the prevalence and effects of anti-abortion religious discourse in the United States is needed and may help abortion advocates counteract these arguments and develop their own messaging for religious audiences.

Our analysis had several limitations. We were unable to collect self-reported demographic information of anti-abortion proponents other than the legislators, so our estimation of participants' demographic characteristics is based on our perceptions of their race, gender, and age, which may be inaccurate. Furthermore, this analysis focused only on anti-abortion arguments, and further research is needed to analyze pro-abortion rhetoric used by those opposing abortion restrictions.

## 5. Conclusions

Restricting abortions has detrimental impacts on the health and well-being of people who can become pregnant (43). At this moment when reproductive rights are being stripped away in the United States, analyses of the discourse and strategies used to promote abortion restrictions are critical. In the current study, we found that arguments used during public legislative hearings to promote a 6-week abortion ban in South Carolina were characterized by scientific disinformation and stigmatizing language around the morality of abortion. More research is needed to determine the optimal tactics to counter this rhetoric; However, advocates for reproductive rights, health, and justice may use findings on current anti-abortion tactics to inform their own strategies, including by explaining to the public and policymakers that current anti-abortion rhetoric is extremely harmful and inaccurate. As the struggle for reproductive rights and justice continues in the United States, abortion advocates must continue to document the dangerous strategies used by abortion opponents and learn from the global pro-abortion movement to develop strategies that will restore these rights.

## Data Availability

Publicly available datasets were analyzed in this study. This data can be found here: Video files of the hearings were downloaded from the Women's Rights and Empowerment Network's Facebook page (https://www.facebook.com/WomensRightsandEmpowermentNetwork) and the South Carolina legislature video archives website (https://www.scstatehouse.gov/video/archives.php).

## Footnotes

^1^These estimates are based both on video footage as well as familiarity the third co-author has with the anti-abortion proponents providing testimony, based on her decades-long participation in abortion access hearings in the SC legislature.

^2^A SC law passed in 2016 (S.C. CODE ANN. § 44-41-330(A)(1)(a)) requires people seeking abortions who have an ultrasound be given the opportunity to see their ultrasound image. However, it does not mandate that people seeking abortions have an ultrasound or view the ultrasound image if they do have one.
